# Resistance of Hepatitis C Virus to Inhibitors: Complexity and Clinical Implications

**DOI:** 10.3390/v7112902

**Published:** 2015-11-06

**Authors:** Celia Perales, Josep Quer, Josep Gregori, Juan Ignacio Esteban, Esteban Domingo

**Affiliations:** 1 Liver Unit, Internal Medicine, Laboratory of Malalties Hepàtiques, Vall d’Hebron Institut de Recerca-Hospital Universitari Vall d’Hebron (VHIR-HUVH), Universitat Autònoma de Barcelona, 08035 Barcelona, Spain; josep.quer@vhir.org (J.Q.); josep.gregori@vhir.org (J.G.); jignacio.esteban@ciberehd.org (J.I.E.); 2 Centro de Biologia Molecular “Severo Ochoa” (CSIC-UAM), Cantoblanco, 28049 Madrid, Spain; edomingo@cbm.csic.es; 3 Centro de Investigación Biomédica en Red de Enfermedades Hepáticas y Digestivas (CIBERehd), 08035 Barcelona, Spain; 4 Universitat Autònoma de Barcelona, Bellaterra 08193, Spain; 5 Roche Diagnostics SL, 08174 Sant Cugat del Vallès, Spain

**Keywords:** viral quasi-species, hepatitis C virus, viral resistance, antiviral treatment

## Abstract

Selection of inhibitor-resistant viral mutants is universal for viruses that display quasi-species dynamics, and hepatitis C virus (HCV) is no exception. Here we review recent results on drug resistance in HCV, with emphasis on resistance to the newly-developed, directly-acting antiviral agents, as they are increasingly employed in the clinic. We put the experimental observations in the context of quasi-species dynamics, in particular what the genetic and phenotypic barriers to resistance mean in terms of exploration of sequence space while HCV replicates in the liver of infected patients or in cell culture. Strategies to diminish the probability of viral breakthrough during treatment are briefly outlined.

## 1. Quasi-Species in the Interpretation of Virus Adaptability

Virologists define quasi-species as dynamic collections of viral genomes subjected to genetic variation, competition, and selection, and which may act collectively as a unit of selection. This definition arose as a consequence of the comparison of nucleotide sequences of individual genomes present in RNA viral populations, and modification of mutant composition as a function of time, both in cell culture and *in vivo*. Molecular or biological clones isolated from a viral population deviated on average in one to several positions from many other genomes of the same population and from the consensus sequence. The consensus or average sequence is the one that at each position has the most abundant residue at that position in the aligned sequences. The imprint of classical genetics, the way sequence data banks have been built, and the inertia of textbook contents, all push to identify viral genomes with a defined nucleotide sequence. The reality is that due to low fidelity replication, populations of RNA viruses—as well as several DNA viruses—consist of mutant clouds rather than a defined sequence. A viral genome can only be defined statistically as a weighted average of many different sequences at any given time, but the precise composition is indeterminate and keeps changing as the virus replicates. Mutation is permanent, not occasional. An identical consensus sequence in successive isolates of a viral lineage does not imply absence of mutations; it means that despite mutations occurring continuously, the variant genomes give rise to the same average genomic sequence. The indeterminacy of the precise composition of a viral population at any time is a resource that viruses exploit for adaptation. It translates into clinical uncertainties such as how important it is to characterize minority genomes before implementing an antiviral protocol, or how will a virus population respond to a given treatment, among other issues, some of which are addressed in coming sections.

The term “unit of selection” included in the quasi-species definition deserves a comment. Viruses subjected to error-prone replication are endowed with a double potential regarding their response to a selective constraint: selection can act on an individual class of genomes (with the same nucleotide sequence) or it may act on a set of genomes (with related nucleotide sequences). In the first case, a single genome or genome class endowed with the adequate phenotype may be selected, irrespective of other genomes around it. However, in their subsequent replication the selected genomes will immediately generate an expanded mutant cloud. In the second case, it is an interconnected ensemble of individuals which is selected at the same time. This dual potential of viral populations for individual or group selection represents by itself a selective advantage over other types of organisms whose populations do not interact internally to provide collective phenotypes as the target of selection (as an overview of quasi-species as applied to viruses and other biological systems, see [1,2,3,4]).

Application of massive parallel sequencing technologies, also known as next generation sequencing (NGS), has confirmed and extended the previous evidence based on molecular or biological cloning and Sanger sequencing that viral populations are indeed very complex, with many minority genomes present at least at 1% level, and most probably at lower levels ([5,6,7,8,9,10,11], among other studies). Lower frequency levels are difficult to reach and characterize due to current methodological limitations. Molecular or biological cloning, combined with Sanger sequencing, despite reaching only about a 20% cut-off for mutation abundance, may still be considered a reference methodology on which the basic quasi-species concepts were built (Section 1 and Section 2). It can probe long sequences, albeit with possible biases derived from using either molecular or biological cloning. Molecular cloning may analyze non-infectious genomes while biological cloning may over-represent the most infectious genomes [12,13,14]. The capacity of NGS to retrieve thousands of sequences per sample is conditioned to identifying and pruning technical sequencing errors, an endeavor which is facilitated by increasingly-effective bioinformatics procedures [15,16,17,18]. A combination of tools to eliminate erroneous reads from NGS data is based on the following three general criteria: (1) general single sequence quality control; (2) exclusion of rare chimeric sequences unlikely to belong to true recombinant genomes; (3) setting a final abundance threshold considered above the noise level [5,9,19,20,21,22,23] (additional features of NGS are summarized in Table 1). Sequencing coverage should be especially taken into account since minority variants above 1% can only be considered if the number of reads reaches a minimum (see point four of Table 1).

**Table 1:** Features of NGS which are relevant to the detection of drug resistance mutations.

Observations	References
Plasmids including point mutations might be used to setting up the detection limit of low frequency resistant mutants within the NGS data treatment pipeline.	[5,9,19,20]
Each haplotype or point mutation (amplicon or shotgun strategy, respectively) should be represented at comparable abundance in forward and reverse DNA strands to be considered.	[5,9,19,20,21,22,23]
There is a general consensus to filter out variants below 1%. However, the threshold above which preexisting mutants may impact on treatment response in patients is still under study.	[5,6,7,8,10]
Based on DeLeeneer *et al.*, the minimal number of reads to theoretically detect a resistant mutant at 1% (observed not below 0.5%) should be 3250. Experimentally, Thys *et al.*, increased to 10,000 reads the minimal coverage to reliably detect minor variants spiked at 1%.	[9,24]
Reconstruction of full-length viral haplotypes is essential to study interactions among mutations present in the same viral RNA molecule. Sequencing of long reads based on single molecule, real-time technology will permit to define such associations.	[25]

The application of NGS to viral quasi-species is forcing a redefinition of what is meant by “conserved” sequences. The traditional invariance of consensus sequences cannot be equated with absence of minority mutations (that have no effect on the consensus) at the relevant region. This is not trivial since conservation in the classical sense (but not in a NGS resolution sense) is the point of departure of designs that claim universal antiviral agents or universal vaccines as objective. The number of publications that deal with the analysis of viral population complexity is increasing at a considerable rate, and the reader is referred to a recent article and two books where the state of the art is summarized, and where many previous studies are quoted [3,4,26].

The collections of genomes that compose viral populations are also termed mutant spectra, mutant clouds or mutant swarms. The population structure denoted by these terms is not only based on definitive experimental evidence, but has a conceptual origin in quasi-species theory developed by M. Eigen and P. Schuster [27,28]. This theory had as its original purpose to explain self-organization and adaptability of primitive genetic elements endowed with inheritable information that might have provided the first replicating forms on Earth. Quasi-species theory is presently an active field of theoretical research, and it has greatly influenced our understanding of basic principles of living systems, including several aspects of virus biology [4]. The core mathematical equation of quasi-species dynamics is not strictly unique. since it can be related to other equations that describe evolutionary dynamics [29]. These relationships among alternative mathematical formulations have one important qualification: the quasi-species equation incorporates mutation as an event inherent to replication. This is not a trivial departure from the other formulations, at least as viruses are concerned, since error-prone replication begs a prominent role of mutation. Additionally, again as an inheritance of classic population genetics, a rooted concept was that mutation was a rare occurrence among present day organisms, with little impact in their present evolution (see [3] for an overview). The inherent association of mutation with replication included in quasi-species theory changed the picture dramatically.

With regard to biological implications of quasi-species dynamics, they can be summarized by saying that quasi-species may impact any viral feature whose performance may vary significantly as a result of one or a few mutations. Very few individual mutations represented in a mutant spectrum will confer genomes the capacity to respond to a specific selective constraint, but the myriads of mutations gathered in a large ensemble will provide a reservoir capable of responding to multiple constraints. Therefore, both mutant spectrum complexity and viral population size are determinants of adaptability. Here we come to the connection between quasi-species and drug resistance, the focus of the present article.

## 2. Molecular Basis of Quasi-Species Dynamics in Connection with Antiviral Drug Resistance

Mutant spectra originate from high mutation rates that, for RNA viruses, have been calculated to be in the range of 10^−3^ to 10^−5^ mutations introduced per nucleotide copied, using biochemical and genetic procedures. The low fidelity of viral polymerases is due to two main molecular mechanisms: (i) the inherently limited accuracy of nucleotide incorporation due to steric requirements during template-directed nucleotide uptake at the polymerase active site; and (ii) the absence of proofreading and post-replicative repair activities. Mechanism (i) is currently being explored at an increasingly-accurate level of atomic resolution, thanks to combined structural and biochemical approaches to the enzymology of viral replication (see as examples [30] and [31], and references therein). Concerning mechanism (ii), the 3′ to 5′ exonuclease activities that can excise incorrect nucleotides incorporated at the 3′-end of the growing polynucleotide chain are absent in most viral RNA polymerases, reverse transcriptases, and some cellular DNA polymerases. In consequence, misincorporated nucleotides remain in the newly-synthesized viral nucleic acids. The post-replicative repair activities operate during cellular DNA, but not viral RNA, replication, because they recognize mismatches in double stranded DNA but not in double stranded RNA or DNA-RNA hybrids. From the enzymology of DNA replication, a similar range of mutation rates is expected for DNA viruses whose replication is catalyzed by polymerases devoid of proofreading-repair activities, although mismatched nucleotides in DNA duplexes may be corrected [32]. It is not clear if post-replicative repair activities are available in sufficient amounts to the replication factories where replicating viral DNAs accumulate, and to what extent such activities diminish progeny heterogeneity [3]. Thus, fundamental biochemical features of mutant generation fit the conceptual postulates of quasi-species theory [4].

In a strict sense according to theory, the term viral quasi-species should refer to the ensemble of genomes present in a single replicative unit; for example, at a replication complex inside an infected cell where a direct dynamics of mutation, competition, and selection occurs. However, the term quasi-species is used in a more relaxed manner to refer to viral genomes that may replicate in different cells, tissues, or organs. This extension is justified since selective constraints and the ensuing competition among viral variants can affect viruses that did not necessarily arise in the same replicative unit. As an example, virus mutants in blood as a result of a systemic infection may be differentially neutralized by antibodies or bind to antiviral agents to different extents, thereby modulating which subpopulations will participate in subsequent replication rounds. These selective processes can be best understood under the quasi-species framework (see [13] for medical implications of viruses replicating as mutant swarms in infected individuals).

To better understand the connection between quasi-species and drug resistance, it is helpful to visualize short-term evolution of viruses as movements in sequence space [13,33,34]. Sequence space is a theoretical representation of all possible sequences available to a polymeric structure. In the case of viral genomes, the total sequence space equals the length in nucleotides to the power of four, since four are the types of monomeric units (nucleotides) that compose the genome. The theoretical sequence space for any virus is an unimaginably large number. However, the portion of sequence space compatible with virus replication, despite being large, is far more reduced, and it is in that portion where events related to adaptability occur. An intuitively useful way to depict the behavior of dynamic mutant clouds is to consider that they are continuously exploring neighbor positions of sequence space, pushed by mutation and guided by fitness (differential rate of multiplication of genome subsets). Each point (or set of points) in sequence space is associated with a relative replicative performance in the face of environmental requirements [35].

The above description is also adequate to capture what “selection” means in viral quasi-species: the replacement of some mutant swarm subpopulations by others, at a rate that depends on the intensity of selection (the classic selection coefficient). Host cell tropism (sometimes with consequences for host range modifications) is only one of many selective pressures that guide movements of mutant clouds in sequence space. Molecular evidence suggests that fitness landscapes (the variation of fitness value as a function of position in sequence space or the environment) are extremely rugged for viruses [3]. The capacity to rapidly explore sequence space is a means to survive in the face of such ruggedness. Furthermore, important features predicted by quasi-species theory—such as the existence of an error threshold for maintenance of genetic information, the basis of the antiviral strategy known as lethal mutagenesis [13]—are maintained for rugged fitness landscape [35].

One of the traits that can be easily selected is drug resistance. Next we address the origins of drug resistance, first in general terms, and then for hepatitis C virus (HCV), at a time at which anti-HCV therapies experience a drastic change through the introduction of many new specific antiviral agents. Current developments with HCV offer an interesting counterpart to the extensive experience gained with antiretroviral agents used to control HIV-1 infections, and the consequences of implementing highly active antiretroviral therapies (HAART) since 1996.

## 3. Molecular and Population Aspects of drug Resistance

Drug- (or inhibitor-) resistant viral mutants are defined as those that can multiply more efficiently than other components of the mutant spectrum in the presence of a drug (or inhibitor). Their selection and maintenance in the viral population is influenced by host and viral factors. Among the latter, six main parameters deserve a comment: (i) the average mutation rate during viral genome replication; (ii) the replication rate that relates to the capacity of exploration of the sequence space; (iii) the viral population size that measures the sequence space actually occupied by the virus; (iv) the genetic barrier to drug resistance that depends on the number and types of mutations needed to attain the resistance phenotype; (v) the phenotypic barrier to resistance that can be quantified as the fitness cost inflicted upon the virus by the resistance mutations; and (vi) the prior evolutionary history of the virus that may have fixed mutations that alter drug sensitivity or barriers to resistance. Most of these parameters are connected, and their influence in the development of drug resistance is summarized in the following paragraphs.

The occurrence of mutations that increase viral replication in the presence of a drug is a random event and, as such, its frequency depends on how many genomes can mutate (the viral population size), how fast they mutate (the mutation rate), and how fast they replicate (their net increase in number as a function of time). These parameters are subjected to trade-offs and limitations. In particular, the mutation rate must lie within some boundaries: too low or too high a rate may result in suboptimal adaptability, as evidenced by the deleterious effect that changes in fidelity of template copying often have on viruses [36]. The replication rate is limited by the molecular machinery of initiation of nucleic acid synthesis and chain elongation; a few measurements suggest that a typical viral RNA genome can be completed in 1 to 10 min (reviewed in [3]), which means efficient progeny production and exploration of sequence space. A consequence of the effect of these parameters is that suboptimal concentrations of inhibitors at the sites of viral replication will favor selection of inhibitor-resistant mutants through maintenance of a replicative load in the presence of the selective agent. Suboptimal inhibitor concentrations can be due to patients not adhering to the treatment schedule, often resulting in treatment failure [37].

For any possible mutant (including drug-resistant mutants) that deviates from the dominant sequence with optimal fitness (the master dominant sequence), the lower its fitness, the lower its frequency in the mutant spectrum (ignoring possible effects of intra-quasi-species complementation or interference). This is a basic principle of quasi-species dynamics, namely that the genomes in a mutant spectrum are ranked according to fitness. If a resistance mutation inflicts a high fitness cost, the relevant mutant will be found at low frequency, and it may not have a chance to become dominant even in the presence of the drug, unless compensatory fitness-enhancing mutations are introduced in the genomes. An unsolved issue is whether low frequency variants which lie at the limit of detection of deep sequencing analyses are still able to replicate at low levels, thereby maintaining a possibility of acquisition of compensatory mutations, or they remain as dead-end genomes unable to evolve. Furthermore, mutant swarms are not mere aggregates of mutant genomes fueled by mutational pressure. Internal interactions of complementation or interference may be established that may enhance or decrease, respectively, the proportion of individual variants, relative to what might have been expected from their fitness measured in isolation (reviewed in [3,4]).

Both the short-term and long-term evolutionary histories are an important influence on drug resistance. The occurrence of a bottleneck (drastic reduction of virus population size) may transiently diminish population complexity and the probability that a specific mutation is found in the population. Long term diversification of viruses in nature, which is defined as a fixation of variations of the dominant nucleotide sequences, leads to increasing numbers of genotypes (GTs) and subtypes that may differ in drug sensitivity or in the barriers to resistance (examples for HCV are given in Section 4.2).

These introductory remarks of drug resistance viewed in the light of quasi-species dynamics renders unsurprising that drug-escape mutants have been systematically described for pathogenic viruses, ever since the first inhibitors became available [38,39]. The ultimate reason is error-prone replication, which is shared by most pathogenic RNA (and several DNA) viruses (additional studies on antiviral resistance have been reviewed in [13,40,41]).

## 4. Resistance to Anti-HCV Inhibitors and Treatment Failure

HCV infections may generate a chronic, progressive disease (chronic hepatitis C, or CHC) that, in most cases, leads to cirrhosis, and constitutes an indication of liver transplantation. Persistent suppression of viral replication can positively affect the course of the infection by impeding disease progression. No vaccine to prevent HCV infection is available, and treatment relies on pharmacological interventions that have seen a profound revolution during the last decade. Yet, the problem of drug resistance and ensuing treatment failures continue being a major difficulty, as examined next for the different historical and current treatment options.

### 4.1. Resistance to Interferon and Ribavirin

The first treatment for patients with CHC became available 30 years ago, and consisted of interferon-alfa-2b (IFN-α-2b). Due to side effects (flu-like symptoms and neuropsychiatric alterations), the adherence to this first treatment was limited, resulting in frequent virus breakthrough. The IFN-only therapy failed to produce a sustained virological response (SVR) in most patients, with SVR rates around 20% [42,43]. Resistance to IFN is a complex trait probably because it requires multiple mutations throughout the HCV genome. According to model studies in cell culture, IFN-α resistance is the result of a complex acquisition of non-synonymous adaptive mutations scattered throughout the genome, accompanied with an increase of the shut-off of host cell protein synthesis, and enhancement of phosphorylation of protein kinase R (PKR) and translation initiation factor eIF2α [44]. The multiple cellular products involved in the IFN response may explain why no clear picture of IFN-α resistance mutations *in vivo* is currently available.

The addition of the purine analogue ribavirin (RBV) to IFN therapy significantly improved long-term virological response in treatment-naïve and treatment-experienced patients [45,46], reaching SVR rates of around 40% [47]. The introduction of pegylated IFN-α (pegIFN-α2a or 2b), used in combination with a body weight-adjusted RBV dose, provided improvements in both efficacy and administration schedule [47]. This combination (abbreviated as pegIFN + RBV) became the standard-of-care regimen for HCV therapy until 2011 [47,48]. Treatment adherence was still limited due not only to IFN-α but also to reversible hemolytic anemia produced by RBV. Numerous clinical trials revealed significantly different response rates that were dependent not only on drug dosage and treatment duration, but on a number of host (allelic forms of some genes) and viral factors, in particular the viral GT. SVR rates ranged from 45% to 93% depending on the viral GT, with the following order of treatment efficacy: GT2 ≈ GT3 ≈ GT5 ≈ GT6 > GT4 ≥ GT1 [49,50,51,52,53].

The molecular basis of the benefits due to inclusion of RBV in the treatment is not well understood [54]. Several antiviral mechanisms of RBV have been described: (i) immunomodulation and enhancement of the Th1 antiviral immune response; (ii) up-regulation of genes involved in IFN signaling; (iii) inhibition of viral RNA-dependent RNA polymerases; (iv) depletion of intracellular GTP levels; (v) inhibition of mRNA cap formation; and (vi) lethal mutagenesis. Several lines of evidence suggest that lethal mutagenesis is involved in the RBV-mediated viral inhibition during anti-HCV therapy [55,56,57,58,59]. The mutagenic activity of RBV has been observed both *in vivo* [57] and in cell culture [60], including a RBV-induced bias in the mutant spectrum which implies an excess of G → A and C → U transitions. In general, selection of a resistance mutation against a classical inhibitor is easier than for a mutagen [3]. The first identification of a RBV-resistance mutation (F415Y in NS5B) in HCV was described in patients under RBV monotherapy [61]. Resistance was also observed in HCV replicon-containing cell lines, and it occurred through changes in the cell line or mutations in NS5A (G404S and E442G). Reduced drug uptake has been proposed as a mechanism for RBV resistance [62,63]. Additionally, serial passage of a GT2a replicon in the presence of RBV resulted in reduced sensitivity to the drug that was associated with NS5B mutation Y33H, apparently due to a reduction in replicative fitness [64]. Passage of infectious J6/JFH1 chimeric HCV in the presence of RBV resulted in a resistant virus, although the mutations responsible for resistance were not identified [65].

### 4.2. Resistance To Directly Acting Antiviral Agents and Host-Targeting Agents

**Figure 1:**
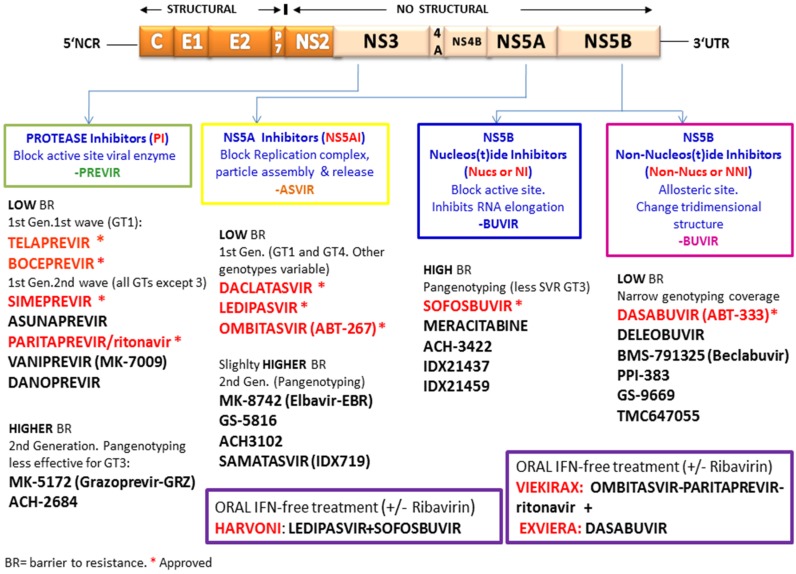
Directly acting antivirals currently available for treatment of hepatitis C virus. Inhibitors target the NS3/4A protease, the non-structural protein NS5A, and the viral polymerase NS5B. Boxes indicate new oral IFN-free combinations.

Since 2011, a new generation of anti-HCV agents, termed Directly-Acting Antivirals (or DAAs) entered the picture of anti-HCV therapy, resulting in great improvement of SVR rates. These new inhibitors target the NS3/4A protease, the non-structural protein NS5A or the viral polymerase NS5B [66,67]. With the introduction of the first-generation HCV NS3/4A protease inhibitors (PI), telaprevir (TPV), and boceprevir (BOC), which are given in combination with pegIFN + RBV, the SVR rates have significantly increased by more than 30%. However, in 20% to 40% of patients, treatment fails and viral load reappears either during therapy (“breakthrough”), or upon treatment interruption (“relapse”). More recently, the approval of new DAAs, such as simeprevir (directed to NS3/4A), daclatasvir (DCV)(directed to NS5A), and sofosbuvir (SOF)(directed to NS5B), as well as oral IFN-free combinations such as ledipasvir/SOF (Harvoni) (directed to NS5A and NS5B, respectively) and triple therapy paritaprevir/ritonavir + ombitasvir + dasabuvir (Viekirax and Exviera) (directed to NS3, NS5A, and NS5B, respectively) have increased the SVR rate to more than 90% in clinical trials with treatment-naïve and cirrhotic patients [67,68,69,70,71,72] (Figure 1).

Despite the potent, and highly-efficient, new treatment regimens, response data outside clinical trials suggest that treatment for around 10% to 15% of patients will fail. A recent study in Denmark showed that only 47% of the patients treated with a triple therapy including TPV or BOC in combination with peg-IFN-α + RBV achieved SVR [73]. This percentage is much lower than that attained in clinical trials. From the patients failing therapy, 71% included mutations that confer resistance to PIs. Data from both *in vitro* analysis and from clinical trials have reliably identified mutations that are associated with treatment failure (reviewed in [74,75]).

Many single amino acid changes are associated with reduced sensitivity to NS3/4A and NS5A inhibitors being the barrier to resistance relatively low [76,77]. Resistance of HCV to nearly all protease inhibitors is achieved by substitutions at key positions in NS3 (Arg 155, Ala 156, and Asp 168) [77,78,79]. Substitutions at positions Met 28, Gln 30, Leu 31, Pro 32, and Tyr 93 in NS5A are frequently selected by NS5A inhibitors [80,81,82,83]. In contrast, the barrier to resistance against NS5B nucleoside analogs is relatively high. Nucleoside inhibitors of NS5B are analogs of the polymerase substrates and bind to residues near the active site which is highly-conserved. As an example, SOF is an approved pyrimidine-derived nucleoside analog NS5B inhibitor whose barrier to resistance is relatively high. However, several substitutions in NS5B (L159F/L320F, T179A, S282T/G/C/R, M289L, I293L, C316N, V321A, I434M) have been associated with SOF resistance, and compensatory mutations (such as M343T and H479P) can contribute to fitness gain in the presence of the main resistance mutation S282T [84,85,86,87].

**Figure 2:**
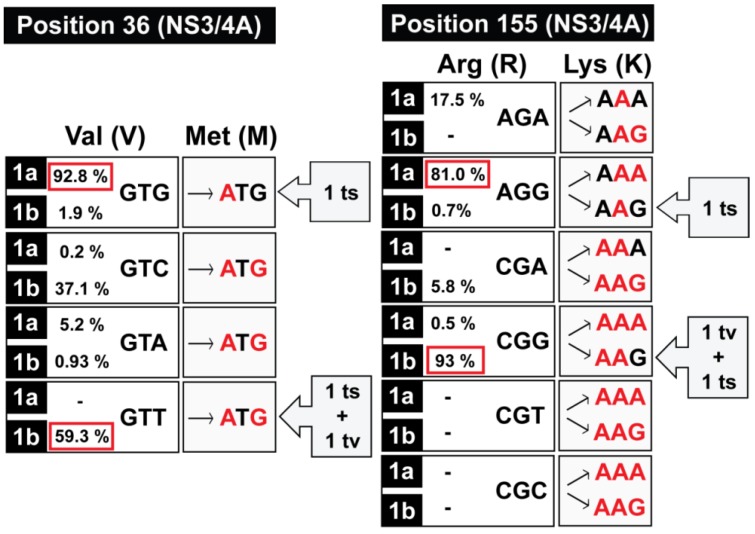
Examples of genetic barrier for generating NS3/4A resistance mutations in HCV. The percentages given for each subtype (1a and 1b) have been calculated with the relevant triplets in a subtype alignment of sequences for GTs 1a and 1b HCV genomic sequences retrieved from [97] with the condition of belonging to full genome sequences, and being devoid of large insertions/deletions (M.E. Soria and C. Perales, unpublished results). The most represented triplets for GTs 1a and 1b are boxed in red; “-“ in the place of a percentage means that the relevant triplet was not represented among the compared sequences. “ts” and “tv” means transition and transversion, respectively. The mutations needed at positions 36 and 155 (resistance to TPV and BOC, among others) are highlighted in red. Data based on several publications quoted in the text.

Substitution S282T has been selected *in vivo* in the course of different treatments that included SOF [88,89,90,91,92]. Additional amino acid changes at position 282 (G/C/R) have been related with SOF resistance using *in silico* models [6], and substitution S282G was also detected in several liver isolates from one patient [93]. HCV offers a direct example of how rapid evolution of a virus in nature and its diversification into GTs and subtypes [94] lead to differences in nucleotide composition that affect the evolutionary pathways towards drug resistance. Several examples of differences in composition of the triplets that encode amino acids in NS3 which participate in inhibitor resistance are given in Figure 2. Such variations can affect the barrier to resistance because of the requirement of either one or two transition or transversion mutations (indicated as ts and tv in Figure 2) to reach the required amino acid. Therefore, virus diversification is, by itself, a source of disparate behavior in terms of inhibitor sensitivities. HCV subtype is a key determinant on the efficacy of DAAs, with subtype 1a patients displaying lower response rates than those infected with subtype 1b [78,95]. A correct subtyping is essential to decide which treatment is the most adequate for each patient and this is now feasible due to NGS technologies [96]. It is still an open question whether resistance to DAAs displaying high barrier to resistance will become a significant clinical issue for treatment failure or not. Experience with other viral diseases suggests that the problem is likely to become relevant. It will largely depend on the management of the new combinations to poorly responding patients—who offer environments that are prone to select for escape mutants—, on the initial fitness capacity of exploration of sequence space for further fitness increases (compare with Section 2), and also on the frequency of transmission of the newly generated mutants.

The difference between resistance to IFN-α and resistance to an antiviral directed against a specific viral target is interesting regarding the different demands on the quasi-species response. Resistance to IFN-α must entail mutations to counteract multiple antiviral components whereas resistance to a DAA is often associated with one or few amino acid replacements, generally in the protein targeted by the inhibitor [98] (Section 4.2). When multiple mutations are needed to overcome an antiviral response such as that induced by IFN-α, the amplitude of the mutant spectrum and the capacity to explore sequence space becomes critical for virus escape (compare with Section 2). That administration of IFN-α alone failed to clear HCV from many patients may be taken as an indication of the multiple evolutionary resources that viral quasi-species exploit for survival [3].

An alternative is the use of designed drugs that target cellular functions required for the virus to complete its infectious cycle, the so called host-targeted agents (HTAs). Since cellular targets cannot vary in response to a viral infection they are an obvious choice to avoid selection of escape mutants [99,100,101,102,103]. Pawlotsky listed 38 host factors involved in the HCV life cycle [104]. The HTAs for HCV that have reached clinical assays or have been licensed for clinical use are inhibitors of cyclophilin A [cyclosporin A (CsA), SCY-635, alisporivir or Debio 025, NIM 811], and miR-122 antagonists. However, two main problems are encountered with the use of HTAs: (i) side effects due to alteration of cellular functions; and (ii) selection of viral escape mutants because the virus can mutate to forms which are capable of by passing the HTA by several mechanisms ([105,106]; reviewed in [3]). It is not clear which will be the contribution of HTAs to anti-HCV combinations displaying a high barrier to resistance.

### 4.3. HCV Inhibitor Resistance Independent of Specific Mutations

A study from our group showed that multiply passaged, high fitness HCV displayed increased resistance to several anti-HCV inhibitors (TPV, DCV, RBV, CsA, and IFN-α) despite the different mechanisms used to exert their antiviral activity. The resistance could not be associated with specific mutations in any case. It was proposed that fitness or a fitness-related trait was responsible of HCV resistance to multiple inhibitors [107]. The molecular basis and the possible clinical impact are still under study but we proposed as a possible mechanism a competition between the number of replicating genomes at each replicative unit in an infected cell and the concentration of inhibitor that reaches the replicative units [107]. Experiments are in progress to explore this possibility. Regarding the possible clinical significance of a fitness-dependent resistance in HCV, some patients do not respond to treatment despite no evidence of resistance substitutions in the expected target protein of the rebound HCV [90,108,109]; several mechanisms have been proposed to account for these observations. If a fitness-related mechanism of HCV resistance operated *in vivo*, some treatment failures would be expected independently of the epidemiological relevance that specific resistance mutations attain in the human population. One of the problems is the difficulty of estimating HCV fitness as the virus replicates *in vivo*. It cannot be excluded that mechanisms of drug resistance exist that are dependent neither on specific mutations nor on a fitness-related trait. This is an important question that has been raised with the new HCV treatments and that was not evident in the follow-up of treatments of other viral diseases.

### 4.4. DAA Resistance Mutations in Naïve and Treatment-Experienced Patients: Effect of Natural and Selected Resistance Mutations on Treatment Response

Two questions are open regarding the influence of resistance mutations in the efficacy of HCV therapy. First, how naturally occurring resistance mutations in viral quasi-species from DAA treatment-naïve patients can affect treatment resolution; and second, how resistance mutations that have been selected as a consequence of a mutational pressure (previous failed treatments) will impact future treatments with the same or a different antiviral drug [74].

With respect to the first question several studies have demonstrated that mutations conferring resistance to NS3/4A, NS5A, or NS5B inhibitors can pre-exist in HCV-infected patients that have not been previously treated with DAAs [110,111,112,113,114,115]. This is not surprising due to the high basal mutation frequency in natural HCV populations. Currently, guidelines from the most important medical associations (EASL and AASLD) only recommend the analysis of substitutions Q80K in NS3/4A in naïve patients due to the high prevalence of this naturally-occurring variant for GT1a and its clear association with decreased SVR after simeprevir-based triple therapy [116,117,118]. Increased failure of the simeprevir-based triple therapy was observed after re-treatment of patients who had developed simeprevir resistance during a previous monotherapy phase, which may be a consequence of the persistence of resistance variants within the viral population [119]. We suspect that, as it happened with antiretroviral therapies for HIV-1, analysis of multiple resistance mutations will be recommended soon.

It is not clear which is the frequency of resistance mutations that can influence the treatment success. It seems that the answer may depend on several variables, such as the viral GT, and the antiviral combinations used. Regarding the effect of the viral GTs, a study using ultra deep pyrosequencing (UDPS) during a long follow-up period in a cohort of patients treated with TPV showed that TPV-resistant variants were amply present at baseline in more than 30% of the patients. Although no general statistically significant differences in the preexistence of resistant mutants between responders and non-responders have been consistently found, the detection of R155K/T/Q at baseline predicted the failure for GT1a patients [115]. On the contrary, the analysis by UDPS of NS3/4A and NS5B regions from 208 DAA-naïve patients infected with GT1a and 1b showed that the prevalence of DAA-resistant mutants and the intrinsic genetic diversity was similar in both isolates suggesting that the composition of the viral quasi-species might not be related to the lower virological response of GT1a as compared to GT1b [112]. Other studies have reported differences in the prevalence of resistant mutants depending not only on the genotype but also on the HCV target region. For instance, population sequencing at baseline in 406 GT1 patients revealed no NS3/4A resistance mutations in GT1b whereas resistance mutations were present in 45% of the GT1a sequences; the frequency of resistance substitutions in NS5A *versus* NS5B depended on the viral subtype (15% and 25% for GT1a and 1b patients in NS5A, and 5% and 28% for GT1a and 1b patients in NS5B) [120].

Regarding the second question or how previously selected resistance mutations can impact future treatments, studies of mutant dynamics during quasi-species evolution in cell culture and *in vivo* suggest that such previously selected mutations may be archived in the form of memory genomes even in viruses that lack a DNA reservoir [121,122,123]. Memory is a consequence of quasi-species behaving as complex adaptive systems, as follows. When a subpopulation of genomes from a viral quasi-species is selected (for example by replicating in the presence of a drug), its proportion in the population increases; because selection entails replication, its fitness increases. When the selective pressure is removed (by eliminating the drug), the selected variants lose dominance in the population. However, because the genomes gained fitness during the selective step, their level in the mutant spectrum is higher than the original level (prior to selection) that was dictated by mutational pressure and basal fitness (compare with the ranking of mutant spectrum components according to fitness discussed in Section 3). When resistant mutants emerge and lead to treatment failure in HCV-infected patients, it is not well known how long they may remain in the population and impact future therapy. Some studies have revealed a loss of detection of resistant variants [108,124], while in other studies, resistant variants were detected several years after treatment with TPV or BOC [125], and after treatment with combinations including NS5A inhibitors [126]. According to model studies in cell culture, the duration of memory genomes in replicating viral populations depends on the fitness level they had when they were established as memory genomes [127]. The data available do not permit anticipating how fitness levels might affect maintenance of inhibitor resistance mutations in HCV from infected patients, although fitness effects on the persistence of resistance mutations after treatment interruption are likely. It is also noteworthy that some amino acid substitutions may impact resistance to several inhibitors (for example amino acids 155, 156 and 168 of NS3/4A regarding resistance to protease inhibitors). Therefore, memory can also impact the response to inhibitors other than the ones used in the first treatment. Memory has also been documented with HIV-1 *in vivo*, and it may contribute to treatment failure when a patient is treated for a second time with a drug that was already administered in a previous treatment [3,123].

## 5. Cell Culture Systems for the Study of Resistance Mutations

The understanding of fundamental processes in virology, including population dynamics, has traditionally been based in combinations of observations in cell culture and *in vivo*. In the case of HCV, having cell culture systems to examine virus replication has been particularly arduous. The first HCV cell culture system that allowed an efficient HCV intracellular replication was achieved in 1999 by R. Bartenschlager’s laboratory [128]. Drug development against HCV, especially DAAs, has been intimately linked to the improvements in the subgenomic replicon system. Despite a strictly intracellular replication, these replicons contain the main targets for therapy (spanning the NS3/4A protease to the NS5B polymerase coding regions), and open the way to the characterization of viral resistance to DAAs [129,130]. As an example, the identification of BMS-790052 (former name of DCV) was based on a high throughput drug screening using replicons [131]. The study of the entire HCV life cycle was possible with the observation that the wild-type JFH1 genome of GT2a produced infectious particles in cell culture [132,133,134]. Based on that, many cell culture systems including JFH1-based chimeras have been developed. The first recombinant viruses were constructed by replacing the core-NS2 region from the seven major GTs [135,136,137,138,139,140,141]. These constructions permitted the analysis of the inhibitory activity of neutralizing antibodies and the screening of inhibitors targeting the core-NS2 [137,142,143]. One step forward in the development of GT-specific JFH1-based chimeras has been the construction of HCV recombinants with sequences encoding the non-structural proteins from NS3/4A to NS5A of different GTs [144,145,146,147,148,149,150]. These cell culture systems have opened the possibility to study not only new aspects of the HCV biology (such as the determination of genetic elements in the non-structural proteins that are similar or that vary among GTs), but also the evaluation of the GT-specific efficacy of antiviral compounds directed against non-structural proteins. These investigations are expected to be extended as the GT-specific cell culture systems are improving, or cell cultures with increased HCV permissivity are developed [151].

The efficacy of NS3/4A protease inhibitors such as TPV, BOC, danoprevir, and vaniprevir was evaluated *in vitro* using recombinant infectious cell culture systems expressing GT-specific NS3 (2a, 3a, 5a, and 6a). The efficacy of protease inhibitors was similar against GTs2a, 5a, and 6a and was lower for GT3a [144]. NS5A is a main target of current DAAs and has been implicated in several viral functions including replication and viral production. The identification of functional domains within the NS5A protein that are important for all GTs might provide a means to develop antiviral compounds active across the spectrum of GTs [146]. The sensitivity to DCV of NS5A-recombinant viruses from GTs one to seven varied depending on the GT that provided the NS5A-coding region, suggesting that GT and subtype are main determinants of the efficacy of specific inhibitors. Conversely, the efficacy of IFN-α treatment was similar with all NS5A-chimeras tested, suggesting that the complete NS5A gene is not sufficient to generate a GT-specific response to IFN-α in cell culture [145]. This is consistent with the multigenic nature of IFN-α resistance in HCV, as emphasized in Section 4.

To evaluate the GT-specific efficacy of combination treatments using NS3 and NS5A inhibitors in the context of the complete viral life cycle, GT1a and 3a recombinants containing only NS3 helicase, NS5B, and the 3′ untranslated region from JFH1 were constructed. The concentrations of asunaprevir (NS3 protease inhibitor) and DCV (NS5A inhibitor) needed to inhibit GT3a viruses were higher than for GT1a. However, combination therapies of both compounds resulted in the suppression of viral infectivity in all recombinants at relatively low concentrations [148].

The construction of molecular clones from GTs different from 2a that are able to produce infectious viruses at high virus titers is one of the major challenges. The main interest for HCV research is in GT1 since a large proportion of HCV infections worldwide are due to that GT. The production of the GT1a infectious virus in cell culture has been improved from the Hutchinson strain [152] to more highly efficient cell culture strains [153,154]; these systems represent a great tool to assess resistance to protease inhibitors [155]. GT1b HCV strains that are able to produce infectious particles have been also described [156,157].

The current view of HCV therapy that we have conveyed is not very distant from the conclusion reached after 30 years of HIV-1 therapy. It will be extremely interesting to observe if new therapies will allow the global eradication of HCV, a pathogen for which no silent DNA reservoirs are known.

## 6. Concluding Remarks and Perspectives

The introduction of DAAs in anti-HCV therapy offers hopes of unprecedented, elevated SVR rates. Yet the problem of drug resistance and treatment failure continues being a limitation. The history of waves of treatment options for HIV-1 infection revives for the hepatitis viruses, in particular HCV. It should not be forgotten that there are still treatment protocols that await exploration. The standard combination therapies may now be applied in modified ways that may further increase SVR rates [158]. These new designs include two-phase split treatments, use of broad spectrum antiviral agents based on stimulation of the innate immune response, or lethal mutagens—that achieve virus extinction by an excess of deleterious mutations—introductions of drugs that target cellular functions needed by the virus, or combinations of immunotherapy and pharmacology. In addition, when a mutagenic agent participates in therapy (as is perhaps the case of RBV as part of its anti-HCV activity) a sequential inhibitor-mutagen combination may have an advantage over the corresponding combination. These several possibilities have been opened as a result of theoretical and experimental studies. It is beyond the scope of the present review article to cover them but the reader will find the main points in some recent publication from our group [3,13,159].

These new possibilities respond to an increasing demand of new paradigms for the control of infectious disease generally, and particularly the challenge of quasi-species swarms. Even if SVRs for HCV treatment reached values close to 100% (a possibility still to be substantiated) due to unprecedented economic investment, improved antiviral interventions will still be needed for a number of emerging and reemerging diseases that are currently expanding as a result of environmental changes, the perfect scenario for viral quasi-species to practice their adaptive capabilities.
